# Effects of combined training with different intensities on vascular health in patients with type 2 diabetes: a 1-year randomized controlled trial

**DOI:** 10.1186/s12933-019-0840-2

**Published:** 2019-03-18

**Authors:** João P. Magalhães, Xavier Melo, Inês R. Correia, Rogério T. Ribeiro, João Raposo, Hélder Dores, Manuel Bicho, Luís B. Sardinha

**Affiliations:** 1 Exercise and Health Laboratory, CIPER, Faculdade de Motricidade Humana, Universidade de Lisboa, Estrada da Costa, 1499-002 Cruz-Quebrada, Portugal; 2 Education and Research Centre, APDP-Diabetes Portugal (APDP-ERC), Lisbon, Portugal; 3 Military Forces Hospital, Lisbon, Portugal; 4 Light Hospital, Lisbon, Portugal; 5 NOVA Medical School, Lisbon, Portugal; 6 Genetics Laboratory Environmental Health Institute (ISAMB), Faculty of Medicine, University of Lisbon, Lisbon, Portugal; 7 Institute of Scientific Research Bento da Rocha Cabral, Lisbon, Portugal; 8 GCP Lab, Ginásio Clube Português, Lisbon, Portugal

**Keywords:** High-intensity interval training, Arterial stiffness, Moderate continuous training, Intima-media thickness, Distensibility coefficient, Pulse wave velocity

## Abstract

**Background:**

Exercise, when performed on a regular basis, is a well-accepted strategy to improve vascular function in patients with type 2 diabetes. However, the exercise intensity that yields maximal adaptations on structural and functional indices in patients with type 2 diabetes remains uncertain. Our objective was to analyze the impact of a 1-year randomized controlled trial of combined high-intensity interval training (HIIT) with resistance training (RT) vs. a combined moderate continuous training (MCT) with RT on structural and functional arterial indices in patients with type 2 diabetes.

**Methods:**

Patients with type 2 diabetes (n = 80) were randomized into an exercise intervention with three groups: control, combined HIIT with RT and combined MCT with RT. The 1-year intervention had 3 weekly exercise sessions. High-resolution ultrasonography of the common carotid artery and central and peripheral applanation tonometry were used to assess the changes in structural and functional arterial indices. Generalized estimating equations were used to model the corresponding outcomes.

**Results:**

After adjusting the models for sex, baseline moderate-to-vigorous physical activity, and mean arterial pressure changes, while using the intention-to-treat analysis, a significant interaction was observed on the carotid intima-media thickness (cIMT) for both the MCT (β = − 4.25, p < 0.01) and HIIT group (β = − 3.61, p < 0.01). However, only the HIIT observed favorable changes from baseline to 1-year on peripheral arterial stiffness indices such as carotid radial arterial pulse wave velocity (β = − 0.10, p = 0.044), carotid to distal posterior tibial artery pulse wave velocity (β = − 0.14, p < 0.01), and on the distensibility coefficient (β = − 0.00, p < 0.01). No effect was found for hemodynamic variables after the intervention.

**Conclusions:**

Following a 1-year intervention in patients with type 2 diabetes, both the MCT and HIIT group reduced their cIMT, whereas only the HIIT group improved their peripheral arterial stiffness indices and distensibility coefficient. Taken together, HIIT may be a meaningful tool to improve long-term vascular complications in type 2 diabetes.

*Trial registration* clinicaltrials.gov ID: NCT03144505

## Introduction

For most patients with diabetes mellitus, micro- and macro-vascular complications are the major culprit for increased mortality and morbidity [1]. With the onset of diabetes, subclinical manifestations of cardiovascular pathology occur, which in turn may lead to ischemic heart disease and carotid stenosis [2, 3]. Among the subclinical markers, carotid intima-media thickness (cIMT) and pulse wave velocity (PWV) are well established structural [4] and arterial stiffness measurements [5], associated with cardiovascular disease (CVD) risk [6, 7]. Patients with type 2 diabetes have increased cIMT and PWV when compared with non-diabetic patients [5, 8]. Prevention and treatment of these markers would likely reverse or delay the vascular changes that occur in type 2 diabetes, improving endpoint outcomes [9, 10].

Current guidelines for patients with type 2 diabetes largely advocate the importance of physical activity as a therapeutic tool to improve and control several CVD risk factors including glycemic control, blood pressure, and endothelium function. In line with these findings, the American Diabetes Association (ADA) states that patients with type 2 diabetes should accumulate at least 150 min of moderate-intensity physical activity [40–60% peak oxygen uptake or 75 min of vigorous-intensity physical activity (60–85% peak oxygen uptake)] per week to maintain or improve health [11, 12]. More recently, studies have reported that performing high intensity interval training (HIIT) can elicit the same or even additional cardiovascular and glycemic adaptations when compared with moderate continuous training (MCT) [13, 14].

While our recent trial [15] suggested that a combination of HIIT with RT had no impact on body composition and glycemic control variables following a 1-year intervention, the effect of HIIT on arterial stiffness and structural indices in patients with type 2 diabetes remains to be determined. In fact, when considering the interventions that included patients with type 2 diabetes and analyzed the impact of HIIT on structural and arterial stiffness measurements, we only found a single case addressing these variables with a time span of 12-weeks [16]. Moreover, to the best of our knowledge, there are no investigations comparing the effects of different types of aerobic exercise (HIIT vs. MCT) alone or in combination with resistance training (combined exercise) on arterial stiffness and structural indexes, which are considered the most effective strategies for achieving glycemic control [17].

Therefore, the purpose of the present 1-year randomized controlled trial was to investigate the effects of a combined HIIT and RT vs. a combined MCT and RT on central and peripheral arterial stiffness indices in patients with type 2 diabetes. We hypothesized that 1-year of a combined HIIT and RT protocol will induce more improvements in cIMT, and on central and peripheral PWV, as well as other stiffness measurements, when compared to a combined MCT with RT protocol.

## Materials and methods

### Recruitment process

**Fig. 1:**
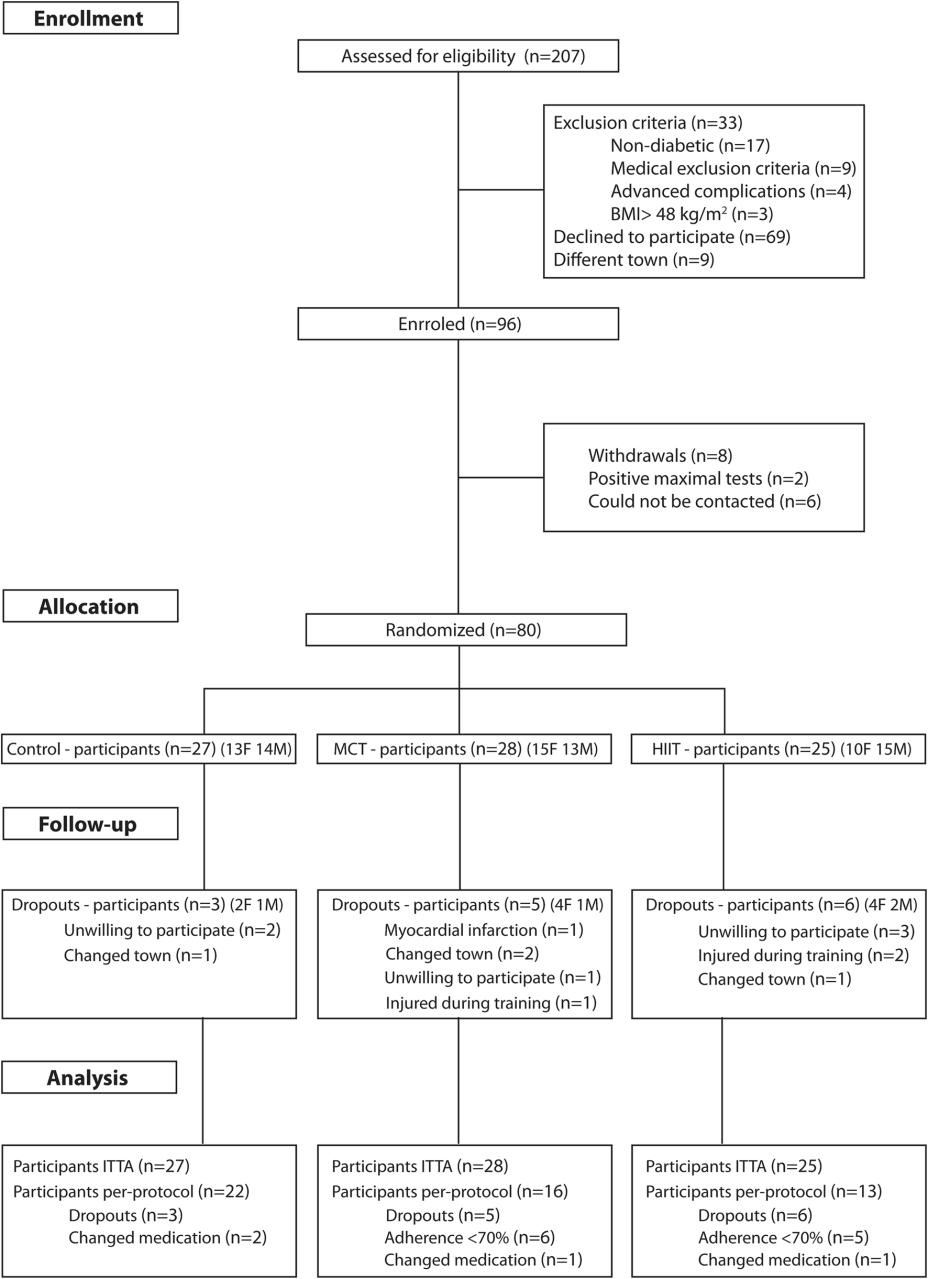
Study flow chart

A randomized controlled trial conducted in the region of greater Lisbon, between February 2014 to July 2016, included patients with type 2 diabetes and an exercise intervention with a 1-year duration (D2FIT project) (Fig. 1). The number and the characteristics of this sample are the same as the one used in our previous published study [15], hence a total of 80 patients were randomized to three distinct groups. In order to be eligible, participants had to be adults diagnosed with type 2 diabetes [18], have no major macro or micro vascular complications, have a body mass index (BMI) < 48 kg/m^2^, and have no physical limitations that would prevent them from participating in an exercise program. Power and sample size calculations (G-Power, Version 3.1.3) were based on a predicted glycated hemoglobin (HbA1c) difference of 0.66 HbA1c units with a SD of effect of 1.2 HbA1c units, α = 0.05, 1 − β = 0.80 and an expected dropout rate of 10% [19]. However, for this secondary analysis the power and sample size calculations were based on changes in aortic PWV. Given a predicted PWV difference of 1.21 m s^−1^ with a SD of 1.4 m s^−1^, α = 0.05, 1 − β = 0.80, the sample used in this study was powered for this secondary analysis [20]. This investigation was carried out in accordance with the recommendations of the Declaration of Helsinki for Human Studies. The protocol was approved by the Ethics Committee of the Portuguese Diabetes Association (approval number: 07/17/2013). Written informed consents were obtained from all participants before and prior to any protocol-specific procedures.

### Research design

The current investigation is a secondary analysis of a 1-year randomized control trial (D2FIT) developed to analyze the impact of exercises with different intensities on glycated hemoglobin (clinicaltrials.gov ID: NCT03144505) [15]. The D2FIT had two intervention groups (MCT with RT, and HIIT with RT) and a control group. An external researcher used a computer-generated list of random numbers, to allocate the patients to the groups in a 1:1:1 ratio. The observers from each assessment were blinded to group randomization.

The changes of arterial stiffness, structural indexes, and hemodynamic variables were assessed as secondary outcomes at baseline and at 1-year follow-up. All evaluations were performed during a single visit, except cardiorespiratory fitness (CRF) assessment, which was evaluated on a separate day. Participants were required to fast for at least 8 h prior to the visit, avoid alcohol consumption for 24 h, and consume a normal evening meal the night before the visit. Participants that were eligible were randomized after completing all the intended assessments.

### Exercise training

The non-exercise group (control) had an initial orientation session, with standard counseling and information regarding general physical activity guidelines. Additionally, once in every 4 weeks, the control group, alongside with the MCT and HIIT group, met for thematic sessions with broad topics such as nutrition, physical activity, and clinical complications from diabetes. All participants from the intervention groups (MCT and HIIT) performed 1-year of supervised exercise sessions three times per week while monitored by certified exercise physiologist and the use of a heart rate polar band (Polar T-31, USA). Both MCT and HIIT were designed to have the same energy expenditure. The duration of the exercise sessions was calculated using a weekly target of 10 kcal/kg and considering individual peak oxygen uptake. For example, considering a patient with a measured VO_2_ peak of 24.2 ml/kg/min, a body weight of 81.2 kg, a standard 3.5 ml/kg/min of resting VO_2_, while exercise at 60% of VO_2reserve_ (15.9 ml/kg/min) during that month, one could expect an energy expenditure of 6.5 kcal/min. When multiplying the body weight for 10 kcal/week we would have 812.5 kcal/week, meaning that at a rate of 6.5 kcal/min, this patient would need to spend 125.5 min/week or 41.8 min of cycling per session. For the HIIT, the intensity calculations were performed with a similar approach as described above, considering that 50% of the exercise sessions were performed at 70–90% of the VO_2reserve_, and the remaining exercise performed at an intensity equivalent to 40–60% of the VO_2reserve_. Considering the example above, this is equivalent to approximately 34 min/session, including 17 HIIT 1-min bouts at 90% of the VO_2reserve_. Throughout the whole year of intervention both groups went through a planned periodization exercise prescription with individualized intensity based upon heart rate reserve (HRR) to gradually introduce the exercise prescriptions. In phase 1 (preparation phase, weeks 1–4), which was similar for both groups, participants performed continuous exercise of moderate intensity (40–60% of the HRR). For the MCT group, which only had an additional phase (training phase, weeks 5–52), participants performed continuous cycling at 40 to 60% of the HRR.

In the HIIT group, the objective of phase 2 (training phase with lower intensity phase, weeks 5–8) was to progressively introduce the HIIT program. During weeks 5–6, participants performed bouts of 2 min at 70% of the HRR followed by 1 min at 40–60% of the HRR and increased to bouts of 80% of the HRR followed by 1 min at 40–60% of the HRR during weeks 7–8. In phase 3 (training phase with higher intensity, weeks 9–52), participants performed 1 min of exercise at 90% of their HRR followed by 1 min resting at 40–60% of the HRR. Throughout the intervention, participants in both groups were instructed to exercise in the upper limit of the prescribed exercise intensity. In both groups, after the aerobic component, participants performed a whole-body RT, which included 1 set of 10–12 repetitions of upper and lower limbs exercises. A full description of the exercise prescription for both MCT and HIIT can be found elsewhere [15].

### Anthropometry

Participants were weighed to the nearest 0.01 kg while wearing minimal clothes and without shoes on an electronic scale (Seca, Hamburg, Germany). Height was measured to the nearest 0.1 cm with a stadiometer (Seca, Hamburg, Germany) according to the standardized procedures described elsewhere [21]. BMI was calculated as body mass (kg)/height^2^ (m). BMI was further categorized into normal (< 25 kg/m^2^), overweight (25–29.9 kg/m^2^), and obese (≥ 30 kg/m^2^). Waist circumference measurement was taken with the participant in a standing position over the naked skin to the nearest 0.1 cm. The tape was applied horizontally just above the uppermost lateral border of the right ilium at the end of normal expiration [22]. The mean of two measurements was considered. If the two measurements differed by more than 1 cm, a third measurement was taken, and the two closest measurements were averaged.

### Brachial blood pressure

The right and left brachial SBP and DBP were measured following at least 15 min with the participants in the supine position using an automated oscillometric cuff (HEM-907-E, Omron, Tokyo, Japan). Two measurements were taken and if these values deviated by > 5 mmHg, a third measurement was performed [23].

### Conduit artery intima-media thickness

The distance between the leading edge of the lumen–intima interface and the leading edge of the media–adventitia interface of the far wall of the right carotid artery was used to define cIMT. This measurement was performed using an ultrasound scanner equipped with a linear 13 MHz probe (MyLab One, Esaote, Italy) [24], and distension curves were acquired within a segment of the carotid artery about one cm before the flow divider. The CV for repeated measurements in our laboratory for cIMT and carotid diameter are 3.02% and 1.49%, respectively.

### Carotid blood pressure

The carotid blood pressure was estimated by assuming a linear relationship with the distension waveform [25], in which the mean arterial pressure (MAP) and brachial DBP are constant over the arterial tree. This enables the calculation of a scaling factor (δ), considering that the integral over time of the distension curve is automatically calculated by the quality arterial stiffness software. The local pressure was assessed using the following formula:

$$ cSBP \left( t \right) = \delta \cdot\Delta D\left( t \right) + bDBP, $$

where ΔD(t) is the distension waveform.

### Carotid arterial stiffness indices

The carotid arterial stiffness measurement was conducted on the right side, with the patient in the supine position after at least a 15 min resting period. An ultrasound scanner equipped with a linear 13 MHz probe (MyLab One, Esaote, Italy) with Quality Arterial Stiffness technology, was used approximately one cm before the bifurcation. This allows the calculation of carotid stiffness indices: PWV (m/s), distensibility coefficient (1/KPa), compliance coefficient (mm^2^/kPa), stiffness index α and β. The coefficients of variation for repeated measurements in our laboratory for carotid distensibility, compliance, stiffness index α and β, and carotid SBP are 0%, 2.87%, 3.70%, 3.18% and 2.15%, respectively.

### Contralateral pulse wave velocity

Pulse wave velocity was measured by applanation tonometry immediately after ultrasound imaging. A single operator located the carotid, femoral, radial, and distal posterior tibial arteries on the left side of the body and marked the point for capturing the corresponding pressure curves with two specific pressure sensitive transducers. The distance between the carotid and femoral and radial and distal posterior tibial arteries were measured directly into the Complior Analyse software (ALAM Medical, Paris, France). PWV values obtained from the carotid to femoral artery (CF PWV), carotid to radial artery (CR PWV), and carotid to distal posterior tibial artery (CD PWV) were taken as indices of central/aortic and peripheral arterial stiffness for upper and lower limb, respectively. A second observer immediately evaluated the quality of the PWV records. Whenever a continuous decrease before the sharp systolic upstroke was not clearly seen, or the tolerance was above 5 ms, a second measure was taken. The coefficients of variation for repeated measurements in our laboratory for aortic, upper limb and lower limb PWV are 2.95%, 9.10%, respectively.

### Physical activity

Participants were asked to wear an accelerometer (Actigraph, model GT1 M model, Pensacola, Florida) on the right hip, near the iliac crest during 4 consecutive days, including 2 weekdays and 2 weekend days. The devices were activated on raw mode with a 100 Hz frequency, and posteriorly downloaded into 15-s epochs (Actilife v.6.9.1). The Troiano et al. cut points and wear time validation criteria were used to define the time spent in each intensity and to define valid measurements [26].

### Cardiorespiratory fitness

Cardiorespiratory fitness was determined using a Bruce standard protocol on a motorized treadmill to exhaustion (model Q-65, Quinton, Cardiac Science Corp; Bothell, WA, USA). All graded exercise tests were monitored using a 12-lead electrocardiogram PC-based acquisition module (model Quark C12, Cosmed, Rome, Italy) with Cosmed software (Cosmed, Rome, Italy). Inspired and expired gases were continuously analyzed, breath-by-breath, through a portable gas analyzer (K4b2, Cosmed, Rome, Italy). Participants exercised until at least two of the following test termination criteria were reached: (1) participants volitional fatigue; (2) respiratory exchange ratio reached 1.1 or higher; (3) participants reached predicted maximal heart rate; (4) oxygen uptake did not increase in spite of increasing workload. The highest 20 s value for peak oxygen consumption (ml/kg/min) attained in the last minute was used in the analysis, and termed CRF from here on.

### Laboratory measurements

Participants underwent biochemical assessments, including the analysis of glycated hemoglobin (HbA1c). Blood samples were collected from an indwelling catheter and drawn into chilled, heparinized tubes and centrifuged rapidly to avoid glycolysis. HbA1c was analyzed by immunoassay (auto analyzer Hb9210 Premier).

### Statistical analysis

The data that support the findings of this study are available from the corresponding author upon reasonable request. Data analyses were performed using IBM SPSS Statistics version 22.0 (SPSS Inc., an IBM Company, Chicago, Illinois, USA). Descriptive statistics including mean ± SD were calculated for all the main and secondary outcome variables. Normality was tested using Q–Q plots. Comparisons between groups were performed using independent sample ANOVA tests or the non-parametric Mann–Whitney–Wilcoxon approach. Due to the correlated nature of the data and repeated measurements taken on each assessment, generalized estimating equations were used to model outcomes while allowing us to control for potential confounders (i.e. sex, baseline moderate-to-vigorous physical activity (MVPA), MAP changes). A least significant difference post hoc test was used to estimate the between-group and within-group effects on several cardiometabolic outcomes. A linear distribution for the response was assumed and an autoregressive correlation matrix was set to the data. For all the outcomes an intention-to-treat analysis (ITTA) was performed using all participants that were initially randomized. Since the D2FIT was designed as an effectiveness investigation, an additional per-protocol analyses (PPA) was performed, which included only a subgroup of participants that completed the investigation and those in the intervention groups that had at least 70% of adherence to the total number of trainings [27].

## Results

Baseline descriptive characteristics of the participants are reported in Table 1 by intervention group and for the ITTA and PPA. The prevalence of overweight and obesity was 83.8%, with 46.3% of the total sample having uncontrolled HbA1c, as defined by ADA criteria (> 7%).

**Table 1:** Baseline characteristics of the participants by group in the ITTA and in the PPA

	Intention to treat baseline values (values are presented mean ± SD)				Per-protocol baseline values (values are presented mean ± SD)			
	Control (n = 27)	MCT (n = 28)	HIIT (n = 25)	p-value	Control (n = 22)	MCT (n = 16)	HIIT (n = 13)	p-value
Age (yrs)	59.0 ± 8.1	59.7 ± 6.5	56.7 ± 8.3	0.575	60.8 ± 7.5	60.4 ± 6.8	58.9 ± 7.5	0.814
Woman (%)	48.1	53.6	40.0	0.612	50.0	56.3	30.8	0.367
Hypertension medication (%)	48.1	50.0	52.0	0.579	54.5	37.5	53.8	0.538
Oral antidiabetic medication (%)	96.3	92.9	84.0	0.388	95.5	93.8	92.3	0.512
Diabetes Diagnosis (yrs)^a^	5.0 ± 3.0	8.0 ± 9.0	5.0 ± 6.0	0.086	4.5 ± 3.25	8.0 ± 9.0	6.0 ± 6.0	0.091
Weight (kg)	84.1 ± 15.8	82.7 ± 13.3	81.6 ± 16.8	0.906	85.9 ± 15.7	82.0 ± 13.8	84.2 ± 19.2	0.799
Height (cm)	165.5 ± 9.4	163.2 ± 8.4	164.8 ± 8.1	0.545	164.5 ± 9.5	162.9 ± 9.2	166.6 ± 8.1	0.615
BMI (kg/m^2^)	30.7 ± 5.0	31.1 ± 5.0	30.1 ± 5.7	0.722	31.7 ± 4.7	31.0 ± 5.5	30.2 ± 5.9	0.506
WC (cm)	103.0 ± 12.4	103.8 ± 11.3	102.7 ± 14.3	0.914	103.3 ± 15.8	103.5 ± 12.2	103.3 ± 15.8	0.946
MVPA (min/day)^a^	18.4 ± 26.4	30.5 ± 4.8	38.9 ± 29.4	0.008*	15.9 ± 23.1	38.1 ± 41.6	38.9 ± 30.1	0.012*
VO_2peak_ (ml/kg/min)	25.9 ± 5.5	24.1 ± 3.2	27.1 ± 6.3	0.143	25.1 ± 5.6	23.9 ± 3.7	26.6 ± 5.3	0.345
HbA1c (mmol/mol)^a^	49.7 ± 20.7	53.2 ± 22.7	49.0 ± 12.3	0.545	48.1 ± 16.7	47.2 ± 22.1	50.8 ± 12.6	0.828

*MCT* moderate continuous training, *HIIT* high intensity interval training, *BMI* body mass index, *MVPA* moderate-to-vigorous physical activity, *HbA1c* glycated hemoglobin, *WC* waist circumference, *VO*_*2peak*_ peak oxygen uptake

* Differences between group at baseline values (p < 0.05)

^a^Skewed values are presented as median ± inter quartile range

At baseline there were no differences between the intervention groups, in both the ITTA and PPA, except for baseline MVPA, which was higher in the HIIT group. A total of 55 participants completed the intervention, with mean adherence percentage values of 86% and 87% for the MCT and HIIT group, respectively. The dropout rates were 11%, 18%, and 24% for the control, MCT and HIIT, respectively (Fig. 1).

Table 2 presents the PPA results for hemodynamic variables and structural and arterial stiffness indices at baseline and following 12 months, as well as the interaction effect of time with each group (MCT vs. HIIT vs. control). Following adjustments for baseline MVPA, sex and MAP, we found an interaction effect for the cIMT in both the MCT (β = − 5.02, p = 0.032) and the HIIT (β = − 3.68, p = 0.045) intervention groups vs. the control group. However, for the distensibility coefficient (β = 0.00, p = 0.0.46) and CD PWV (β = − 0.20, p > 0.01), only the HIIT group had a significant interaction effect following a 1-year of intervention. Hemodynamic variables, as assessed by brachial blood pressure and carotid blood pressure, did not change throughout the intervention in both exercise groups. Following the same trend, we observed no interaction effect for the CF PWV for all intervention groups (p > 0.05).

**Table 2:** Hemodynamic, structural and functional arterial indices at baseline and following 12 months: within and between group changes using the per-protocol analysis

Outcome	Control (n = 22)		MCT (n = 16)		HIIT (n = 13)		MCT*Control	HIIT*Control	MCT*HIIT
	Baseline	12 months	Baseline	12 months	Baseline	12 months	β (95% CI)	β (95% CI)	β (95% CI)
SBP (mmHg)	136.5 ± 12.6	130.6 ± 21.4	135.6 ± 13.6	130.9 ± 19.2	137.8 ± 17.0	130.7 ± 16.1^†^	0.08 (− 0.79; 0.94)	0.09 (− 0.73; 0.90)	− 0.01 (− 0.85; 0.83)
DBP (mmHg)	80.1 ± 9.3	77.4 ± 10.8	81.2 ± 9.6	78.2 ± 9.1	80.2 ± 9.1	74.5 ± 6.3^†^	− 0.01 (− 0.50; 0.47)	− 0.22 (− 0.61; 0.16)	0.21 (− 0.29; 0.72)
Carotid IMT (mm)	714.9 ± 130.7	751.2 ± 119.4^†^	737.9 ± 158.4	712.3 ± 110.8	733.6 ± 159.0	724.1 ± 119.2	− 5.04 (− 9.57; − 0.50)*	− 3.70 (− 7.38; − 0.01)*	− 1.34 (− 6.62; 3.93)
Carotid DC	0.018 ± 0.007	0.015 ± 0.007	0.019 ± 0.009	0.018 ± 0.009	0.018 ± 0.006	0.019 ± 0.007	0.00 (− 0.00; 0.00)	0.00 (0.00; 0.00)*	0.00 (− 0.00; 0.00)
Carotid β SI	11.9 ± 4.5	14.2 ± 6.8^†^	11.2 ± 4.1	12.3 ± 4.9	11.9 ± 5.1	12.6 ± 6.2	− 0.11 (− 0.31; 0.09)	− 0.15 (− 0.35; 0.06)	0.04 (− 0.19; 0.27)
Carotid SBP (mmHg)	120.9 ± 15.3	117.7 ± 13.9	124.2 ± 13.0	117.2 ± 11.9^†^	123.8 ± 14.3	121.4 ± 12.3	− 0.31 (− 0.85; 0.23)	0.08 (− 0.57; 0.73)	− 0.39 (− 1.07; 0.29)
Carotid DBP (mmHg)	73.7 ± 7.0	70.9 ± 7.7	77.3 ± 9.4	73.1 ± 5.9^†^	75.9 ± 9.3	74.9 ± 6.2	− 0.13 (− 0.55; 0.28)	0.15 (− 0.41; 0.71)	− 0.29 (− 0.84; 0.27)
CF PWV (m/s)	13.1 ± 4.8	14.0 ± 4.3	13.3 ± 3.8	14.3 ± 3.9	12.8 ± 4.0	13.5 ± 4.7	0.01 (− 0.15; 0.18)	− 0.02 (− 0.17; 0.13)	0.03 (− 0.14; 0.19)
CD PWV (m/s)	9.0 ± 1.8	10.3 ± 1.7^†^	9.9 ± 2.0	9.7 ± 1.4	9.7 ± 1.5	8.6 ± 2.0^†^	− 0.12 (− 0.24; 0.00)	− 0.20 (− 0.31; − 0.09)*	0.08 (− 0.03; 0.18)
CR PWV (m/s)	9.2 ± 1.9	9.3 ± 1.5	9.6 ± 1.6	9.0 ± 2.1	9.5 ± 1.4	8.3 ± 1.9	− 0.06 (− 0.18; 0.06)	− 0.11 (− 0.25; 0.03)	0.05 (− 0.11; 0.20)
VO_2peak_ (ml/kg/min)	25.5 ± 5.5	24.4 ± 5.7	24.0 ± 3.6	25.1 ± 4.9	28.0 ± 7.0	27.0 ± 6.9	0.21 (0.05; 0.37) *	0.02 (− 0.13; 0.17)	0.19 (0.04; 0.35)*

Betas are presented as unstandardized coefficients adjusted for sex, baseline MVPA, and Map changes, with the respective 95% confidence intervals

*DC* distensibility coefficient, *CD PWV* carotid distal pulse wave velocity, *CF PWV* carotid femoral pulse wave velocity, *CR PWV* carotid radial pulse wave velocity, *DBP* diastolic blood pressure, *HIIT* high intensity interval training, *IMT* intima media thickness, *MCT* moderate continuous training, *SBP* systolic blood pressure, *SI* stiffness index, *VO*_*2peak*_ peak oxygen uptake

*Between-group changes significant at p < 0.05

^†^Within-group changes significant at p < 0.05

**Fig. 2:**
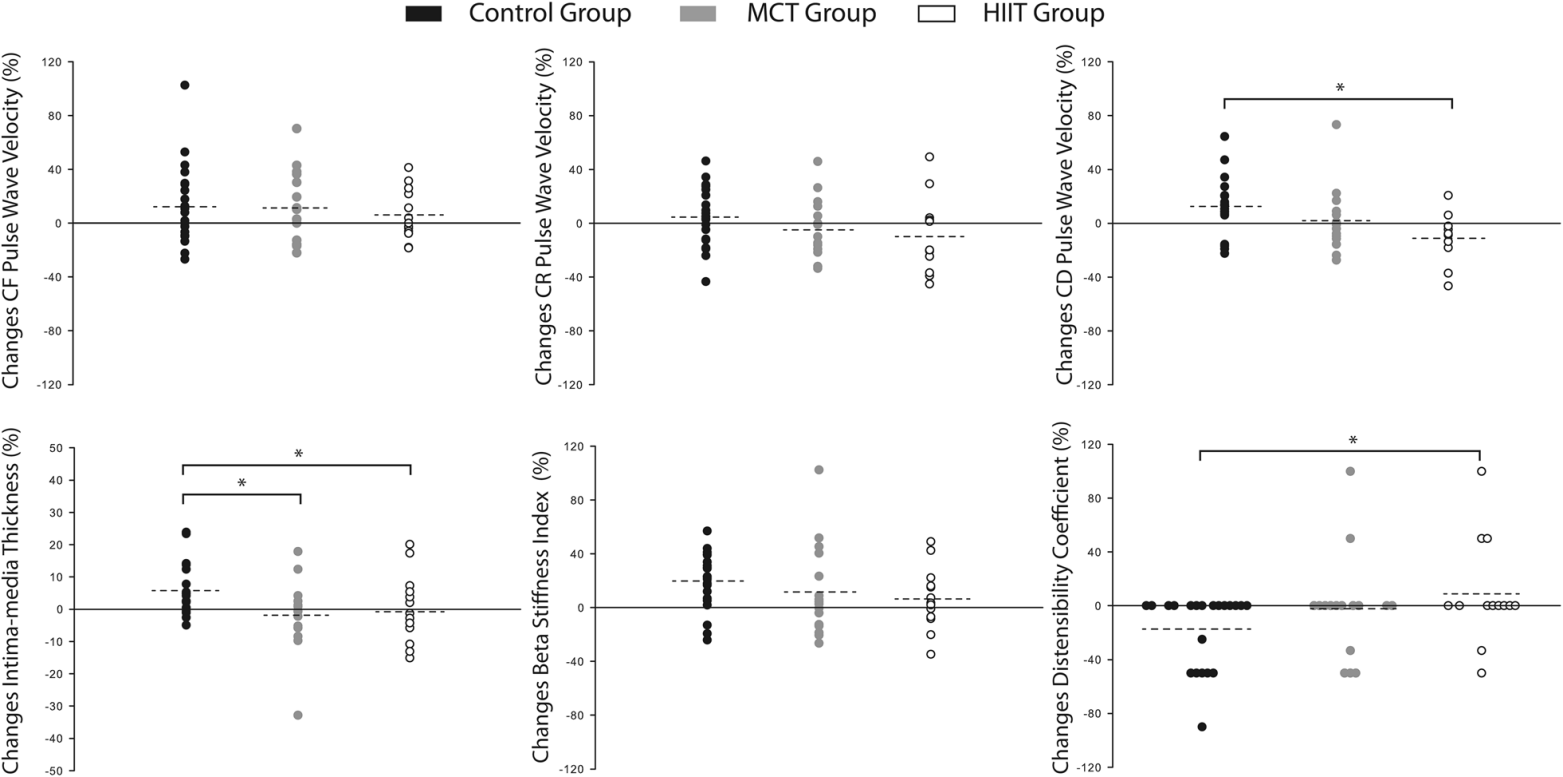
Changes in structural and hemodynamic variables using the per-protocol analysis. Dashed lines represent mean values for each intervention group

Figure 2 depicts data from the changes that occurred between baseline and 1-year follow-up for the structural and arterial stiffness indices using the PPA. Participants from the HIIT intervention group decreased their cIMT, CR PWV, and CD PWV values by, 1.1%, 10.0% and 11.2%, respectively. With similar favorable changes, the HIIT group also increased the distensibility coefficient by 8.9%. When considering the MCT group, we observed changes only for the cIMT variable, with a decrease of 1.4% from pre- to post-intervention.

Table 3 summarizes the results of the ITTA for hemodynamic variables and structural and arterial stiffness indices. When considering the ITTA, and after adjusting for sex, MAP and baseline MVPA, the results remained similar as the PPA for all the outcomes, with MCT (β = − 4.25, p < 0.01) and HIIT (β = − 3.61, p < 0.01) intervention groups decreasing their cIMT values from baseline to post-intervention. The HIIT group also had a favorable effect on the CD PWV (β = − 0.14, p < 0.01) and on the distensibility coefficient (β = 0.00, p < 0.01), similar to those observed in the PPA, while having an additional effect on CR PWV (β = − 0.10, p = 0.044). As far as all other variables are concerned, no interaction effects were observed for both intervention groups.

**Table 3:** Intention-to-treat analysis on hemodynamic, structural and functional arterial indices

Outcome	Control (n = 22)		MCT (n = 16)		HIIT (n = 13)		MCT*Control	HIIT*Control	MCT*HIIT
	Baseline	12 months	Baseline	12 months	Baseline	12 months	β (95% CI)	β (95% CI)	β (95% CI)
SBP (mmHg)	136.8 ± 13.4	131.5 ± 20.6	139.9 ± 13.5	134.6 ± 18.0	142.2 ± 18.3	135.7 ± 19.3^†^	− 0.04 (− 0.74; 0.66)	0.04 (− 0.65; 0.73)	− 0.08 (− 0.75; 0.60)
DBP (mmHg)	81.2 ± 10.5	77.5 ± 11.1^†^	82.0 ± 8.8	79.3 ± 10.1	82.6 ± 10.3	78.4 ± 10.1^†^	0.07 (− 0.34; 0.48)	− 0.03 (− 0.35; 0.29)	0.10 (− 0.29; 0.49)
Carotid IMT (mm)	716.5 ± 120.6	746.7 ± 109.7^†^	723.1 ± 142.5	703.1 ± 106.8	713.2 ± 175.8	700.2 ± 151.6	− 4.25 (− 7.38; − 1.12)*	− 3.61 (− 6.15; − 1.07)*	− 0.64 (− 4.06; 2.78)
Carotid DC	0.019 ± 0.006	0.016 ± 0.006^†^	0.018 ± 0.008	0.018 ± 0.008	0.017 ± 0.006	0.018 ± 0.006	0.00 (− 0.00; 0.00)	0.00 (0.00; 0.00)*	0.00 (− 0.00; 0.00)
Carotid β SI	11.3 ± 4.2	13.4 ± 6.2^†^	11.7 ± 4.1	12.6 ± 4.8	11.8 ± 4.3	12.2 ± 4.9	− 0.09 (− 0.23; 0.05)	− 0.14 (− 0.28; 0.01)	0.05 (− 0.10; 0.19)
Carotid SBP (mmHg)	121.5 ± 16.0	119.4 ± 14.8	126.2 ± 12.5	121.8 ± 14.7^†^	126.0 ± 17.6	124.0 ± 16.4	− 0.20 (− 0.65; 0.25)	0.01 (− 0.50; 0.52)	− 0.21 (− 0.75; 0.33)
Carotid DBP (mmHg)	75.4 ± 9.0	73.6 ± 9.8	77.7 ± 8.3	74.9 ± 7.3^†^	76.9 ± 8.7	76.4 ± 9.3	− 0.11 (− 0.43; 0.22)	0.12 (− 0.28; 0.51)	− 0.22 (− 0.60; 0.16)
CF PWV (m/s)	12.9 ± 4.4	13.5 ± 4.1	13.0 ± 3.3	14.0 ± 3.5^†^	13.2 ± 3.7	13.9 ± 4.1	0.03 (− 0.09; 0.14)	− 0.00 (− 0.11; 0.10)	0.03 (− 0.07; 0.13)
CD PWV (m/s)	9.2 ± 2.0	10.1 ± 1.8	10.1 ± 1.8	10.1 ± 1.5	10.2 ± 2.7	9.5 ± 2.7	− 0.08 (− 0.17; 0.02)	− 0.14 (− 0.24; − 0.04)*	0.06 (− 0.03; 0.15)
CR PWV (m/s)	8.9 ± 2.3	9.0 ± 2.1	9.4 ± 1.4	9.2 ± 1.7	10.3 ± 2.2	9.3 ± 2.3	− 0.03 (− 0.11; 0.06)	− 0.10 (− 0.19; − 0.00)*	0.07 (− 0.03; 0.17)
VO_2peak_ (ml/kg/min)	25.9 ± 5.5	24.4 ± 5.4	24.1 ± 3.2	24.9 ± 4.1	27.1 ± 6.3	26.5 ± 6.0	0.19 (0.03; 0.34)*	0.05 (− 0.076; 0.64)	0.12 (0.01; 0.22)*

Betas are presented as unstandardized coefficients adjusted for sex, baseline MVPA, and MAP changes, with the respective 95% confidence intervals

*CD* carotid distensibility, *CD PWV* carotid distal pulse wave velocity, *CF PWV* carotid femoral pulse wave velocity, *CR PWV* carotid radial pulse wave velocity, *DBP* diastolic blood pressure, *HIIT* high intensity interval training, *IMT* intima media thickness, *MCT* moderate continuous training, *SBP* systolic blood pressure, *SI* stiffness index, *VO*_*2peak*_ peak oxygen uptake

* Between-group changes significant at p < 0.05

^†^Within-group changes significant at p < 0.05

## Discussion

To our knowledge, this is the first randomized controlled trial spanning a 1-year intervention with supervised exercise sessions analyzing the effectiveness of a MCT combined with RT vs. HIIT combined with RT on structural and functional arterial indices in patients with type 2 diabetes. This investigation affords experimental evidence that a long-term MCT combined with RT and HIIT combined RT improves cIMT. Moreover, the results of the study highlights HIIT as a promising long-term exercise strategy to delay and counter the changes in arterial stiffness indices, more specifically on CD PWV, CR PWV, and on the distensibility coefficient, which are typically compromised in ageing and in type 2 diabetes, with likely implications on the progression of CVD.

Type 2 diabetes is strongly linked to central obesity, which in turn is associated with increased arterial stiffness [28]. Indeed, patients with type 2 diabetes have higher values of arterial stiffness when compared to matched controls [29–31]. Regarding the benefits of HIIT on vascular health in patients with type 2 diabetes, most of the literature available derives from flow mediated dilation (FMD) studies. Recent reviews and meta-analysis have found different results on the benefits of HIIT on FMD in patients with type 2 diabetes when compared to MCT [32–34]. Unlike FMD, PWV provides structural alongside with functional health information of the arterial wall and can be used as a complementary measurement to that of FMD. In our investigation, baseline values for CF PWV and CD PWV were 13.0 ± 4.0 and 9.6 ± 1.9, respectively, which are slightly higher than the ones observed in other studies, especially for the CF PWV [5, 16]. Exercise seems to attenuate arterial stiffness in healthy subjects and in patients with type 2 diabetes, but the effect of different exercise intensities still remain controversial. Epidemiological evidence suggests that the largest impact on vascular risk occurs when practicing exercise at lower volumes and intensities [35, 36]. However, most of the studies included in these meta-analyses and reviews used high-intensity continuous training to analyze the impact of exercise intensity on vascular risk [37]. Engaging in vigorous PA for prolonged periods of time in a continuous fashion results in increased oxidative stress due to the elevated ROS production, which could jeopardize nitric oxide bioavailability and mitigate the positive effects of exercise on the endothelium [36, 38]. By limiting exercise at higher intensities to periods of shorter bouts, HIIT could potentially limit these effects on nitric oxide bioavailability, since the exercise bout is always followed by a recovery period [34]. Nevertheless, notwithstanding the potential role that intensity plays on improving vascular health, the increased energy expenditure derived from physical activity has been associated with a reduced risk of CVD [39].

The D2FIT investigation hopes to shed light into this intensity subject by adding valuable long-term experimental evidence of the effects of different exercise intensities on vascular health in people with type 2 diabetes. Our findings suggest that after a 1-year intervention a reduction in the peripheral arterial stiffness, particularly in the CD PWV (1.1 m/s) and CR PWV (1.2 m/s), was only observed in the HIIT group, but no changes were observed for CF PWV. Central stiffness worsens within groups, but CD PWV tends to improve. Since CF PWV is a component of CD PWV, one can hypothesize that peripheral stiffness improves, perhaps to an even greater extent than is suggested by the CD PWV data alone. Nonetheless, only a reduction in CF PWV is associated with reduced risk of cardiovascular and all-cause mortality [40]. While using a similar HIIT protocol to the one of this investigation, Francois et al. [16], analyzed the effects of HIIT on arterial stiffness in patients with type 2 diabetes. Following a 12-week intervention, the HIIT group observed a reduction of 1.6 m/s in the CF PWV, whereas no changes were observed for CR PWV. The differences between these results and the ones from our investigation might be related with the duration of the intervention as well as the period in which these variables were assessed. The D2FIT investigation was designed as a 1-year intervention that had two time point assessments for the vascular health variables (baseline and 1-year follow-up). Therefore, the results observed after 1-year intervention might not reflect the short (2–4 weeks) and medium-term changes that occur with the exercise training on vascular health, which were not assessed in our investigation due to the lack of mid-point assessments. Nevertheless, a recent meta-analyses that investigated the impact of different exercise modalities on both central and peripheral arterial stiffness in adults, observed that only aerobic exercise had an effect on arterial stiffness, specifically on peripheral (CR PWV and CD PWV) rather than on central indices (CF PWV) [41]. This effect was not significant in patients who used combined MCT with RT, which follows the same results of our intervention.

These results for the aerobic exercise alone might be explained by the properties of peripheral arteries, which are more muscular and stiffer than central, more elastic arteries [36], allowing for their larger adaptations in response to exercise training. Additionally, the HIIT cycling exercise program maximized muscle fiber recruitment through engagement og a larger amount of skeletal muscle mass, which may have promoted adaptations on local muscle fibers and the on blood vessels that supply them [42]. We can speculate that the superior adaptations observed on peripheral stiffness (CD PWV) might be due to the characteristics of the HIIT program, which generated more local adaptations when compared to MCT. As far as the impact of HIIT combined with RT is concerned, there may be an interference effect on arterial stiffness remodeling, favoring exercises performed at higher intensities. Another possible explanation for the superior benefits of HIIT on arterial stiffness, lies on the ability of HIIT to promote an up-regulation of PGC-1α in the local muscle, which is superior to that observed following MCT [43, 44]. Overexpression of PGC-1α in the endothelial cells that have the potential to improve antioxidant defenses of the mitochondria [45]. Therefore, the superior results on the vascular function observed in HIIT, could partly be due to a decrease in ROS, without affecting NO availability [34]. Alongside with the structural changes, the HIIT group also observed an increase in the distensibility coefficient of the carotid artery, whereas no changes were observed for the MCT group. Such results are important for vascular health, since arterial stiffness and arterial dispensability deteriorate with ageing and type 2 diabetes [46, 47].

Beyond the arterial stiffness adaptations, we observed structural changes in the cIMT favoring both the MCT and HIIT groups. cIMT is a well-established marker for predicting future cardiovascular events in patients with type 2 diabetes [48]. The fact that a 1-year intervention promoted a significant reduction in cIMT is important because the carotid artery is highly susceptible to the development of atherosclerosis and is an important marker for identifying patients who are at higher risk of developing micro- and macrovascular complications [49]. To our knowledge, this is the first investigation to analyze which type of exercise induces greater improvements on the cIMT (HIIT vs. MCT). So far, the experimental evidence addressing this topic has focused on randomized control trials with only one type of exercise. Francois et al., with a 12-week intervention using HIIT, found no effect of the exercise intervention on cIMT. The lack of results might be explained by the short duration of the investigation, which may not be enough time to observe structural changes in the intima-media layer. With similar results, a 1-year intervention using a combined MCT with RT protocol also observed no changes in cIMT in patients with type 2 diabetes. However, there was an interaction effect between the exercise group and the patients without atherosclerotic plaques, where patients without early stages of atherosclerotic disease improved their cIMT [50]. The proposed mechanisms mediating the improvements in cIMT suggest that repeated exposure to shear stress, associated with exercise training, represents a key stimulus for arterial remodeling [51]. The importance of the nitric oxide pathway and the lower production of vascular adhesion molecules are also mechanisms through which exercise induced shear stress can improve structural remodeling [51, 52]. Exercise is known to improve hyperglycemia and elevated levels of insulin, which are characteristic of patients with type 2 diabetes and have been shown to impair the production and availability of nitric oxide [42]. A recent review [53], has highlighted the potential of HIIT to improve glycemic control to a greater extent, when compared with MCT, hence providing a potential link between insulin resistance and improved vascular health.

As far as CRF is concerned, and as previously reported [15], we observed favorable changes in CRF, but only in the MCT with RT group. Increased CRF values are strongly associated with improvements in vascular health [54, 55], as well as reductions in CVD development and other type 2 diabetes associated co-morbidities [56, 57]. However, changes in vascular health and on other risk factors are not totally dependent on improvements in CRF. Recently, Pandey et al. observed that patients who failed to improve their CRF following a long-term aerobic exercise and/or resistance training, did experience significant improvements in several risk factors (e.g. glycemic control and body composition) [58]. Thus, it is possible that improvements in vascular health variables may also be independent of improvements in CRF.

The present investigation also has some limitations that warrant discussion. First, the D2FIT investigation had a 1-year follow-up period with only two assessment moments for all the vascular health variables (baseline and at 1-year). Further assessment points would have provided a valuable insight on the progression of structural and functional vascular indices throughout the intervention, especially in the 1st months. Moreover, since this study is part of a secondary analysis it is possible that number of enrolled patients might be reduced to draw significant conclusions on IMT regression after 1 year. Also, owing to HIIT, it is possible that psychological fatigue may have set in towards the final months of the intervention, raising questions of the long-term applicability of this kind of training in patients with type 2 diabetes.

A major strength of the D2FIT investigation was that, unlike previous medium-term investigations [16], D2FIT was an intervention with a 1-year duration designed to analyze the long-term effects of HIIT on vascular health-related variables in patients with type 2 diabetes. Another strength was that the exercise followed a planned periodization and individualized tailored exercise program that was supervised throughout all sessions in an ecological exercise setting. Finally, both exercise groups were designed to be iso-energetic based on the assumption that the total volume of exercise would meet current PA guidelines.

## Conclusions

In conclusion, both MCT and HIIT had favorable effects on structural arterial variables, such as cIMT, after a 1-year exercise intervention in patients with type 2 diabetes. However, only HIIT had an effective impact on peripheral arterial stiffness variables and on local distensibility of the carotid artery, providing experimental evidence to suggest that HIIT can induce clinical meaningful reductions in both structural and functional indices of vascular health after a 1-year intervention. These results may have future implications for exercise interventions in patients with type 2 diabetes with goals of targeting and attenuating the micro- and macrovascular changes inherent to this disease.

## Abbreviations

CRF: cardiorespiratory fitness
cIMT: carotid intima-media thickness
CD PWV: carotid to distal posterior tibial artery pulse wave velocity
CF PWV: carotid to femoral artery pulse wave velocity
CR PWV: carotid to radial artery pulse wave velocity
FMD: flow mediated dilation
HRR: heart rate reserve
HIIT: high intensity interval training
ITTA: intention-to-treat analysis
MAP: mean arterial pressure
MCT: moderate continuous training
MVPA: moderate-to-vigorous physical activity
PPA: per-protocol analyses
PWV: pulse wave velocity

## References

[1] Budoff MJ, Raggi P, Beller GA, Berman DS, Druz RS, Malik S (2016). Noninvasive cardiovascular risk assessment of the asymptomatic diabetic patient: the imaging council of the american college of cardiology. JACC Cardiovasc Imaging.

[2] Eringa EC, Serne EH, Meijer RI, Schalkwijk CG, Houben AJ, Stehouwer CD (2013). Endothelial dysfunction in (pre)diabetes: characteristics, causative mechanisms and pathogenic role in type 2 diabetes. Rev Endocr Metab disord.

[3] Fox CS, Coady S, Sorlie PD, D’Agostino RB, Pencina MJ, Vasan RS (2007). Increasing cardiovascular disease burden due to diabetes mellitus—the Framingham Heart Study. Circulation.

[4] Miyamoto M, Kotani K, Okada K, Ando A, Hasegawa H, Kanai H (2013). Arterial wall elasticity measured using the phased tracking method and atherosclerotic risk factors in patients with type 2 diabetes. J Atheroscler Thromb..

[5] Teoh WL, Price JF, Williamson RM, Payne RA, Van Look LAF, Reynolds RM (2013). Metabolic parameters associated with arterial stiffness in older adults with Type 2 diabetes: the Edinburgh Type 2 Diabetes Study. J Hypertens.

[6] Lorenz MW, Sitzer M, Markus HS, Bots ML, Rosvall M (2007). Prediction of clinical cardiovascular events with carotid intima-media thickness: a systematic review and meta-analysis—response. Circulation.

[7] Mitchell GF, Hwang SJ, Vasan RS, Larson MG, Pencina MJ, Hamburg NM (2010). Arterial stiffness and cardiovascular events the framingham heart study. Circulation.

[8] Kotb NA, Gaber R, Salama M, Nagy HM, Elhendy A (2012). Clinical and biochemical predictors of increased carotid intima-media thickness in overweight and obese adolescents with type 2 diabetes. Diabetes Vasc Dis Res.

[9] Kim SH, Lee SJ, Kang ES, Kang S, Hur KY, Lee HJ (2006). Effects of lifestyle modification on metabolic parameters and carotid intima-media thickness in patients with type 2 diabetes mellitus. Metabolism..

[10] Way KL, Keating SE, Baker MK, Chuter VH, Johnson NA (2016). The effect of exercise on vascular function and stiffness in type 2 diabetes: a systematic review and meta-analysis. Curr Diabetes Rev.

[11] Colberg SR, Albright AL, Blissmer BJ, Braun B, Chasan-Taber L, Fernhall B (2010). Exercise and type 2 diabetes: american college of sports medicine and the american diabetes association: joint position statement. Exercise and type 2 diabetes. Med Sci Sports Exerc.

[12] WHO, Organization WH (2010). WHO global recommendations on physical activity for health. WHO global recommendations on physical activity for health. WHO guidelines approved by the guidelines review committee.

[13] Wormgoor SG, Dalleck LC, Zinn C, Harris NK (2017). Effects of high-intensity interval training on people living with type 2 diabetes: a narrative review. Can J Diabetes..

[14] Batacan RB, Duncan MJ, Dalbo VJ, Tucker PS, Fenning AS (2017). Effects of high-intensity interval training on cardiometabolic health: a systematic review and meta-analysis of intervention studies. Br J Sport Med..

[15] Magalhaes JP, Judice PB, Ribeiro R, Andrade R, Raposo J, Dores H (2018). Effectiveness of high intensity interval training combined with resistance training vs. continuous moderate intensity training combined with resistance training in patients with type 2 diabetes—1 year randomized controlled trial. Diabetes Obes Metab.

[16] Francois ME, Pistawka KJ, Halperin FA, Little JP (2018). Cardiovascular benefits of combined interval training and post-exercise nutrition in type 2 diabetes. J Diabetes Complicat..

[17] Davies MJ, D’Alessio DA, Fradkin J, Kernan WN, Mathieu C, Mingrone G, et al. Management of hyperglycaemia in type 2 diabetes, 2018. A consensus report by the American Diabetes Association (ADA) and the European Association for the Study of Diabetes (EASD). Diabetologia. 2018. 10.1007/s00125-018-4729-5 30288571

[18] Hove-Skovsgaard M, Gaardbo JC, Kolte L, Winding K, Seljeflot I, Svardal A (2017). HIV-infected persons with type 2 diabetes show evidence of endothelial dysfunction and increased inflammation. BMC Infect Dis.

[19] Boule NG, Haddad E, Kenny GP, Wells GA, Sigal RJ (2001). Effects of exercise on glycemic control and body mass in type 2 diabetes mellitus: a meta-analysis of controlled clinical trials. JAMA.

[20] Guimaraes GV, Ciolac EG, Carvalho VO, D’Avila VM, Bortolotto LA, Bocchi EA (2010). Effects of continuous vs. interval exercise training on blood pressure and arterial stiffness in treated hypertension. Hypertens Res.

[21] Lohman TG, Roche AS, Martorell R (1988). Anthropometric standardization reference manual.

[22] CDC. National health and nutrition examination survey (NHANES)—anthropometry procedures manual. https://www.cdc.gov/nchs/nhanes/nhanes2015-2016/manuals15_16.htm2016. Accessed 26 July 2018.

[23] Pickering TG, Hall JE, Appel LJ, Falkner BE, Graves J, Hill MN (2005). Recommendations for blood pressure measurement in humans and experimental animals: part 1: blood pressure measurement in humans: a statement for professionals from the Subcommittee of Professional and Public Education of the American Heart Association Council on High Blood Pressure Research. Circulation.

[24] Hoeks AP, Willekes C, Boutouyrie P, Brands PJ, Willigers JM, Reneman RS (1997). Automated detection of local artery wall thickness based on M-line signal processing. Ultrasound Med Biol.

[25] Van Bortel LM, Balkestein EJ, van der Heijden-Spek JJ, Vanmolkot FH, Staessen JA, Kragten JA (2001). Non-invasive assessment of local arterial pulse pressure: comparison of applanation tonometry and echo-tracking. J Hypertens.

[26] Troiano RP, Berrigan D, Dodd KW, Masse LC, Tilert T, McDowell M (2008). Physical activity in the United States measured by accelerometer. Med Sci Sports Exerc.

[27] Intention to treat analysis and per protocol analysis: complementary information. Prescrire Int. 2012;21(133):304–6. 23373104

[28] Sutton-Tyrrell K, Newman A, Simonsick EM, Havlik R, Pahor M, Lakatta E (2001). Aortic stiffness is associated with visceral adiposity in older adults enrolled in the study of health, aging, and body composition. Hypertension.

[29] Ryden L, Standl E, Bartnik M, den Berghe G, Betteridge J, de Boer MJ (2007). Guidelines on diabetes, pre-diabetes, and cardiovascular diseases: executive summary—The Task Force on Diabetes and Cardiovascular Diseases of the European Society of Cardiology (ESC) and of the European Association for the Study of Diabetes (EASD). Eur Heart J.

[30] Shin JY, Lee HR, Lee DC (2011). Increased arterial stiffness in healthy subjects with high-normal glucose levels and in subjects with pre-diabetes. Cardiovasc Diabetol.

[31] Zhang MH, Bai YY, Ye P, Luo LM, Xiao WK, Wu HM (2011). Type 2 diabetes is associated with increased pulse wave velocity measured at different sites of the arterial system but not augmentation index in a chinese population. Clin Cardiol.

[32] Qiu S, Cai X, Yin H, Sun Z, Zugel M, Steinacker JM (2018). Exercise training and endothelial function in patients with type 2 diabetes: a meta-analysis. Cardiovasc Diabetol..

[33] Miele EM, Headley SAE (2017). The effects of chronic aerobic exercise on cardiovascular risk factors in persons with diabetes mellitus. Curr DiabRep.

[34] Ramos JS, Dalleck LC, Tjonna AE, Beetham KS, Coombes JS (2015). The impact of high-intensity interval training versus moderate-intensity continuous training on vascular function: a systematic review and meta-analysis. Sports Med.

[35] Lee JH, Lee R, Hwang MH, Hamilton MT, Park Y (2018). The effects of exercise on vascular endothelial function in type 2 diabetes: a systematic review and meta-analysis. Diabetol Metab Syndr.

[36] Green DJ, Hopman MTE, Padilla J, Laughlin MH, Thijssen DHJ (2017). Vascular adaptation to exercise in humans: role of hemodynamic stimuli. Physiol Rev.

[37] Goto C, Higashi Y, Kimura M, Noma K, Hara K, Nakagawa K (2003). Effect of different intensities of exercise on endothelium-dependent vasodilation in humans - Role of endothelium-dependent nitric oxide and oxidative stress. Circulation.

[38] Bergholm R, Makimattila S, Valkonen M, Liu ML, Lahdenpera S, Taskinen MR (1999). Intense physical training decreases circulating antioxidants and endothelium-dependent vasodilatation in vivo. Atherosclerosis..

[39] Amadid H, Johansen NB, Bjerregaard AL, Brage S, Faerch K, Lauritzen T (2018). The role of physical activity in the development of first cardiovascular disease event: a tree-structured survival analysis of the Danish ADDITION-PRO cohort. Cardiovasc Diabetol..

[40] Guerin AP, Blacher J, Pannier B, Marchais SJ, Safar ME, London GM (2001). Impact of aortic stiffness attenuation on survival of patients in end-stage renal failure. Circulation.

[41] Ashor AW, Lara J, Siervo M, Celis-Morales C, Mathers JC (2014). Effects of exercise modalities on arterial stiffness and wave reflection: a systematic review and meta-analysis of randomized controlled trials. PLoS ONE.

[42] Olver TD, Laughlin MH (2016). Endurance, interval sprint, and resistance exercise training: impact on microvascular dysfunction in type 2 diabetes. Am J Physiol Heart Circ Physiol.

[43] Wisloff U, Stoylen A, Loennechen JP, Bruvold M, Rognmo O, Haram PM (2007). Superior cardiovascular effect of aerobic interval training versus moderate continuous training in heart failure patients: a randomized study. Circulation.

[44] Tjonna AE, Lee SJ, Rognmo O, Stolen TO, Bye A, Haram PM (2008). Aerobic interval training versus continuous moderate exercise as a treatment for the metabolic syndrome—a pilot study. Circulation.

[45] Valle I, Alvarez-Barrientos A, Arza E, Lamas S, Monsalve M (2005). PGC-1alpha regulates the mitochondrial antioxidant defense system in vascular endothelial cells. Cardiovasc Res.

[46] Taddei S, Virdis A, Mattei P, Ghiadoni L, Gennari A, Fasolo CB (1995). Aging and endothelial function in normotensive subjects and patients with essential-hypertension. Circulation.

[47] Laurent S, Caviezel B, Beck L, Girerd X, Billaud E, Boutouyrie P (1994). Carotid-artery distensibility and distending pressure in hypertensive humans. Hypertension.

[48] Bernard S, Serusclat A, Targe F, Charriere S, Roth O, Beaune J (2005). Incremental predictive value of carotid ultrasonography in the assessment of coronary risk in a cohort of asymptomatic type 2 diabetic subjects. Diabetes Care.

[49] Sibal L, Agarwal SC, Home PD (2011). Carotid intima-media thickness as a surrogate marker of cardiovascular disease in diabetes. Diabetes Metab Syndr Obes Targets Ther.

[50] Byrkjeland R, Stensaeth KH, Anderssen S, Njerve IU, Arnesen H, Seljeflot I (2016). Effects of exercise training on carotid intima-media thickness in patients with type 2 diabetes and coronary artery disease. Influence of carotid plaques. Cardiovasc Diabetol.

[51] Tuttle JL, Nachreiner RD, Bhuller AS, Condict KW, Connors BA, Herring BP (2001). Shear level influences resistance artery remodeling: wall dimensions, cell density, and eNOS expression. Am J Physiol Heart Circ Physiol.

[52] O’Keeffe LM, Muir G, Piterina AV, McGloughlin T (2009). Vascular cell adhesion molecule-1 expression in endothelial cells exposed to physiological coronary wall shear stresses. J Biomech Eng.

[53] Grace A, Chan E, Giallauria F, Graham PL, Smart NA (2017). Clinical outcomes and glycaemic responses to different aerobic exercise training intensities in type II diabetes: a systematic review and meta-analysis. Cardiovasc Diabetol..

[54] Buscemi S, Canino B, Batsis JA, Buscemi C, Calandrino V, Mattina A (2013). Relationships between maximal oxygen uptake and endothelial function in healthy male adults: a preliminary study. Acta Diabetol.

[55] Boreham CA, Ferreira I, Twisk JW, Gallagher AM, Savage MJ, Murray LJ (2004). Cardiorespiratory fitness, physical activity, and arterial stiffness: the Northern Ireland Young Hearts Project. Hypertension.

[56] Church TS, LaMonte MJ, Barlow CE, Blair SN (2005). Cardiorespiratory fitness and body mass index as predictors of cardiovascular disease mortality among men with diabetes. Arch Intern Med.

[57] Katzmarzyk PT, Church TS, Janssen I, Ross R, Blair SN (2005). Metabolic syndrome, obesity, and mortality: impact of cardiorespiratory fitness. Diabetes Care.

[58] Pandey A, Swift DL, McGuire DK, Ayers CR, Neeland IJ, Blair SN (2015). Metabolic effects of exercise training among fitness-nonresponsive patients with type 2 diabetes: the HART-D study. Diabetes Care.

